# Rhizospheric Bacterial Distribution Influencing the Accumulation of Isoflavones, Phenolics, Flavonoids, and Antioxidant Activity in Soybean Roots Within Hydroponic System

**DOI:** 10.3390/plants14142238

**Published:** 2025-07-19

**Authors:** Du Yong Cho, Mu Yeun Jang, Hee Yul Lee, Jong Bin Jeong, Da Hyun Kim, Do Yun Bang, Hye Rim Kim, Ye Rim Jeong, Md. Azizul Haque, Jin Hwan Lee, Kye Man Cho

**Affiliations:** 1 Department of GreenBio Science, Agri-Food Bio Convergence Institute, Gyeongsang National University, Jinju 52727, Republic of Korea; endyd6098@gnu.ac.kr (D.Y.C.); jmy4330@naver.com (M.Y.J.); jjb4421@gnu.ac.kr (J.B.J.); lanelane1004@gmail.com (D.H.K.); bdy@gnu.ac.kr (D.Y.B.); koko012360@naver.com (H.R.K.); dpfl35@gmail.com (Y.R.J.); 2 Gyeongnam Anti-Aging Research Institute, Sancheong-gun 52215, Republic of Korea; hylee0614@gari.or.kr; 3 Department of Biochemistry and Molecular Biology, Hajee Mohammad Danesh Science and Technology University, Dinajpur 5200, Bangladesh; helalbmb2016@hstu.ac.bd; 4 Department of Smart Green Resources, Dong-A University, 37, Nakdong-daero 550 beon-gil, Saha-gu, Busan 49315, Republic of Korea

**Keywords:** soybean roots, hydroponic cultivation, rhizosphere bacteria, isoflavones, antioxidants, hydroponic soybean root bacteria

## Abstract

This study investigates how root color in soybeans affects isoflavone composition, rhizosphere bacterial diversity, total phenolics, total flavonoids, and antioxidant activity under a hydroponic cultivation system. Notably, soybean-brown roots (SBRs) accumulated significantly higher contents of isoflavones, exhibiting approximately a 14.9-fold increase in total glycosides (141.75 to 2121.59 µg/g), 7.3-fold increase in total malonyl-β-glycosides (127.52 to 930.45 µg/g), 2.8-fold increase in total aglycones (1825.90 to 5145.21 µg/g), and 3.9-fold increase in total isoflavones (2095.16 to 8197.26 µg/g) than soybean-white roots (SWRs). Isolated rhizosphere bacteria profiling revealed γ-*Proteobacteria* as the predominant class in both root types, constituting 77.6% and 73.9% of the bacterial community in SWRs and SBRs, respectively. However, SBRs supported a more diverse bacterial ecosystem, harboring thirteen genera compared to only eight genera in SWRs. Enhanced total phenolics, total flavonoids, and radical scavenging activity were also associated with the SBRs. These findings shed light on the dynamic interplay between root traits, bacterial interactions, and secondary metabolite biosynthesis in hydroponically grown soybeans. This work not only advances our understanding of plant root–microbiome–metabolite relationships but also offers a novel approach to exploring the potential of enhancing secondary metabolites in soybean plants through precision cultivation.

## 1. Introduction

Soybeans (*Glycine max*) are well-known sources of isoflavones, which are called phytoestrogens, and have shown numerous health advantages [1]. However, recent studies have revealed that isoflavone derivatives, especially aglycones such as daidzein and genistein, are also enriched in the roots of soybean seedlings [2,3], where they function as key signaling molecules that mediate plant defense mechanisms and shape interactions with rhizospheric bacteria [4]. In particular, isoflavones help in reducing cholesterol levels in humans, consequently lowering the risk of cardiovascular diseases [5]. Furthermore, isoflavones enhance the mineral density in bones, which subsequently lowers the risk of osteoporosis, especially in the postmenopausal phase [6]. Therefore, conceptualizing the intricate relationship between the distribution of rhizospheric bacterial communities and isoflavone enrichment specifically in the root system of soybean seedlings is of particular significance in this study.

The rhizospheric bacterial communities influence the overall health of the plant, including the promotion of nutrient uptake, regulation of growth, suppression of pathogens, and enhancement of tolerance to environmental stresses. [7,8]. Rhizospheric bacteria promote the synthesis of isoflavones in soybean plants, thereby intensifying plants’ defense mechanisms [9]. For example, *Bradyrhizobium* sp. establishes nitrogen-fixing nodules in soybean roots, subsequently regulating the production of isoflavones. These symbiotic interactions contribute to improving plant nutrition and pathogen resistance [9,10]. Additionally, rhizospheric bacteria synthesize plant hormones, auxins, and cytokinins, to promote root elongation and lateral root formation, which increase root surface area and enhance the absorption of water and nutrients [11]. Furthermore, certain bacterial strains facilitate the degradation of soil pollutants and uplift soil fertility, thereby creating a conducive environment for soybean plant growth [12].

The nutrient film technique (NFT) in a hydroponic system ensures flawless access to essential nutrients, oxygen, and water in plant roots, which provide healthy roots with increased plant vigor [13]. The utilization of NFT systems enhances crop yield and quality by markedly reducing infestation of soil-borne diseases and facilitating precise control over environmental conditions [14]. Importantly, the industrial utilization of root zones has traditionally been limited due to their close association with soil, which poses challenges in terms of hygiene, consistency, and scalability [15]. The use of a hydroponic NFT system overcomes these limitations by providing a controllable environment for root production. In particular, the NFT system has demonstrated effectiveness as a method for cultivating soybean crops [16], enabling comprehensive interactions of root morphology and rhizospheric bacterial community [17]. This unique characteristic renders the NFT an invaluable tool in investigating the influence of rhizosphere bacterial distributions on the production of secondary metabolites [18], yielding insights into sustainable agricultural strategies aimed at improving soybean quality.

This study aims to focus on exploring the effects of rhizospheric bacterial distribution on enriching isoflavones in soybean roots by utilizing the NFT in a hydroponic system. We will focus on the differences between soybean-white root (SWR) and soybean-brown root (SBR) to analyze how variations in root morphology relate to rhizospheric bacterial communities. We investigated the potential associations between variations in rhizospheric bacterial distribution between SWR and SBR and the resultant changes in isoflavone accumulation, thereby offering valuable insight into plant–microbe interactions that significantly influence metabolite profiles. Conceptualizing these dynamics will enable us to explore strategies for intensifying the production of physiologically active compounds in soybeans and enhancing their metabolite values.

## 2. Results

### 2.1. Length and Biomass of SWR and SBR

**Figure 1:**
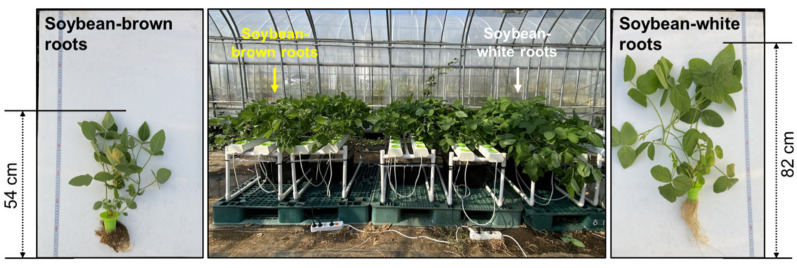
Photograph of the produced soybean plants in a hydroponic cultivation system. Soybean plants were grown in a greenhouse maintained at a temperature of 25 ± 3 °C and humidity of 70 ± 5% for 35 days. Also, they were subirrigated with tap water for the first week and then with nutrient solutions.

The morphological examinations revealed that the SWR, grown in hydroponic conditions within sunny areas of greenhouses, exhibited white roots. In contrast, the SBR, which was grown in hydroponic conditions within semi-shaded areas of greenhouses, exhibited brown roots. The length of the soybean roots is depicted in Figure 1. The length measurements indicated that the SWRs reached a length of 82 cm, while the SBRs recorded a length of only 54 cm. As seen in Figure S1, the fresh weights of leaves were 4.16 and 2.87 g, stems were 3.23 and 2.28 g, and roots were 2.63 and 2.49 g for SWRs and SBRs, respectively. In addition, the dry weights of leaves were 0.34 and 0.27 g, the stems were 0.24 and 0.19 g, and the roots were 0.12 and 0.10 g, in the order of SWR and SBR. Furthermore, the SWR (52.9%) of the number of cultivated plants was a higher percentage than the SBR (47.1%) (No data provided). These results indicated a superior development in the SWR group.

### 2.2. Comparison of Isoflavone Contents in SWR and SBR

**Figure 2:**
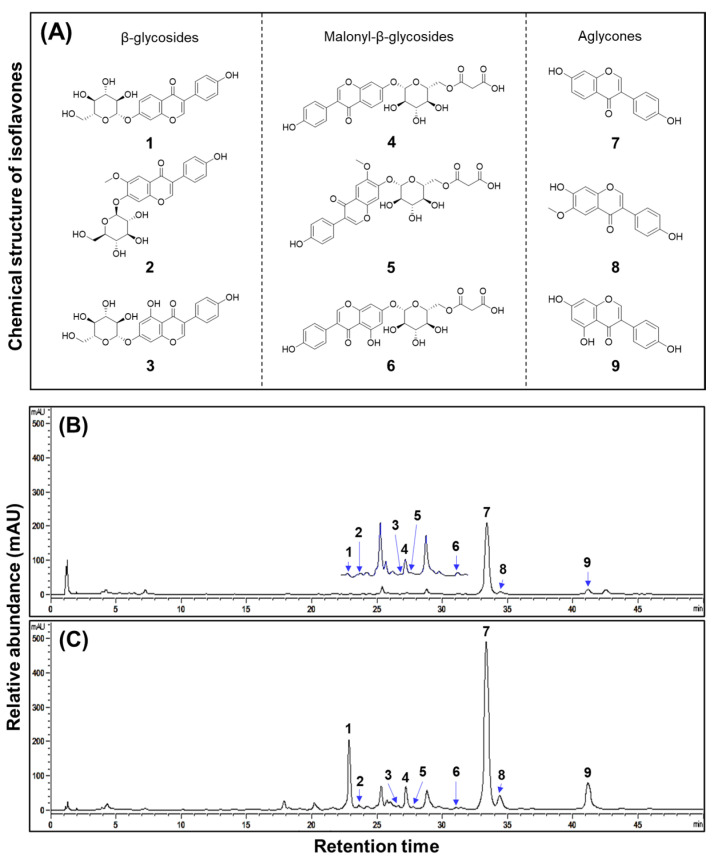
Chemical structure and typical HPLC chromatogram of isoflavones from the produced soybean-white roots and soybean-brown roots in a hydroponic cultivation system. (**A**) Chemical structure of isoflavones; HPLC chromatogram of (**B**) soybean-white roots; and (**C**) soybean-brown roots. 1. β-daidzin; 2. β-glycitin; 3. β-genistin; 4. malonyl-β-daidzin; 5. malonyl-β-glycitin; 6. malonyl-β-genistin; 7. daidzein; 8. glycitein; and 9. genistein.

The isoflavone contents in the organs of two distinct groups of soybean plants (SWR and SBR) cultivated within a hydroponic system were systematically compared and analyzed. A total of nine types of isoflavone derivatives were identified. Statistically significant differences in isoflavone contents were observed among the samples (Figure 2). Notably, daidzein derivatives (β-daidzin, malonyl-β-daidzin, and daidzein) exhibited significantly higher concentrations compared to genistein derivatives (β-genistin, malonyl-β-genistin, and genistein) across root samples, indicating the dominance of the daidzein series within the principal isoflavone composition of soybean roots. When comparing roots of the two groups, SBRs were distinguished by a markedly enhanced concentration of daidzein derivatives relative to SWRs. Specifically, β-daidzin was quantified as 1608.13 μg/g in the SBR sample, which was approximately 17.0-fold higher than in the SWR sample. Moreover, malonyl-β-daidzin was approximately 9.5-fold higher, quantified to be 662.29 μg/g, and daidzein was approximately 2.5-fold higher, quantified at 4035.27 μg/g. The isoflavone contents in the upper parts (leaves and stems) tended to be slightly higher in SBRs than in SWRs. Specifically, the total isoflavone content in SBR leaves (2141.99 µg/g) was slightly higher than that in SWR leaves (1912.59 µg/g), and a similar trend was quantified in the stems (SBR: 621.66 µg/g and SWR: 529.54 µg/g) (Table 1). Nevertheless, these differences were not significant compared to those in the root organs.

### 2.3. Comparison of Bacterial Distribution in SWR and SBR

**Figure 3:**
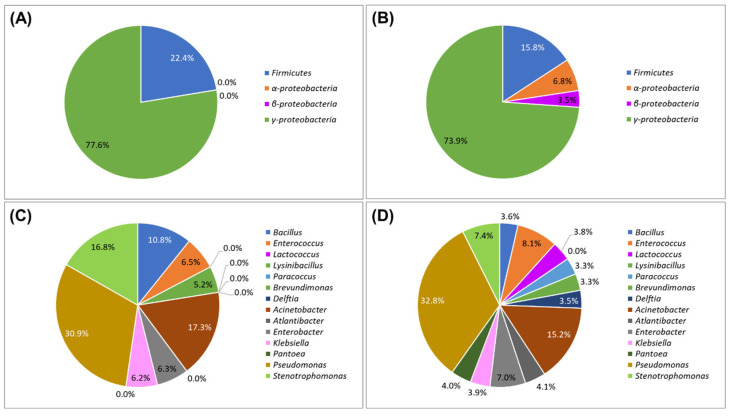
Bacterial distribution of the produced soybean-white roots and soybean-brown roots in the hydroponic cultivation system. (**A**) Phylum of soybean-white roots; (**B**) phylum of soybean-brown roots; (**C**) genus of soybean-white roots; and (**D**) genus of soybean-brown roots.

To elucidate the intricate interactions of the soybean roots with the rhizospheric bacterial community distribution, the community distribution was identified in each SWR and SBR sample. In consequence, a total of seventeen different species of bacteria were identified in SWR samples, while SBR samples revealed a total of twenty-six different bacterial species (Figures S2 and S3, Table 2). In case of SWRs, *Firmicutes* comprised 22.4% at the phylum level, whereas *γ-Proteobacteria* constituted 77.6%, thereby establishing *γ-Proteobacteria* as the predominant rhizospheric bacterial group (Figure 3A). At the genus level, a total of eight genes were identified, with *Pseudomonas* (30.9%), *Acinetobacter* (17.3%), and *Stenotrophomonas* (16.8%) being the most prevalent (Figure 3C). While in the case of SBRs, the detected phyla were *Firmicutes* (15.8%), *α-Proteobacteria* (6.8%), *β-Proteobacteria* (6.8%), and *γ-Proteobacteria* (73.9%), again highlighting the predominance of *γ-Proteobacteria*, while also confirming the presence of additional subgroups within the *Proteobacteria* category (Figure 3B). At the genus level for SBR, thirteen genera were identified, with the major genera being *Pseudomonas* (32.8%), *Acinetobacter* (15.2%), and *Enterococcus* (8.1%) (Figure 3D). Notably, *Lactococcus* (3.8%), *Paracoccus* (3.3%), *Delftia* (3.5%), *Atlantibacter* (4.1%), and *Pantoea* (4.0%) were not identified in SWRs but were present in SBRs.

### 2.4. Comparison of Pearson’s Correlation Coefficient in SWR and SBR

**Figure 4:**
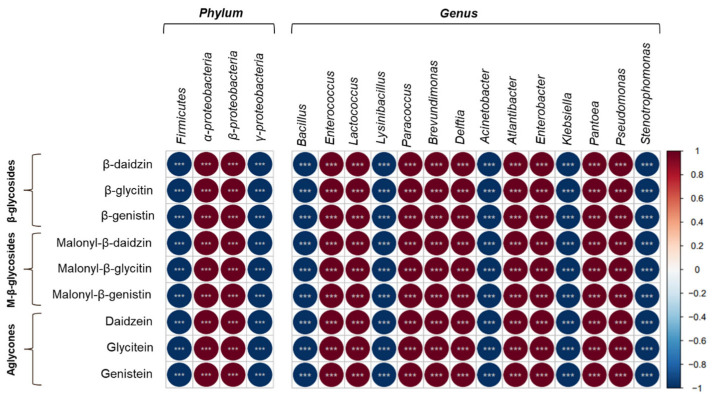
Pearson correlation coefficient between rhizosphere bacteria and root isoflavones of the produced soybean-white roots and soybean-brown roots in the hydroponic cultivation system. Dark red and blue show positive and negative correlations, respectively. ⁎⁎⁎, *p* < 0.001.

Diverse rhizosphere bacteria are present in the roots of the soybean plant, which may be correlated with isoflavone changes. Figure 4 presents the Pearson’s correlation coefficient matrix derived from the relative abundance of bacteria isolated from SWR and SBR samples at both the phylum and genus levels. Initially, at the phylum level, *α-proteobacteria* and *β-proteobacteria* exhibited strong positive correlations with all isoflavone derivatives. Conversely, *Firmicutes* and *γ-proteobacteria* displayed negative correlations, indicating a contrasting trend. At the genus level, *Enterococcus*, *Lactococcus*, *Paracoccus*, *Brevundimonas*, *Delftia*, *Atlantibacter*, *Enterobacter*, *Pantoea*, and *Pseudomonas* demonstrated significant positive correlations with the isoflavone derivatives. Notably, eighteen species that displayed such positive correlations were included among the twenty-six species isolated from SBRs, with *Paracoccus*, *Brevundimonas*, and *Delftia* notably absent in SWRs and exclusively abundant in SBRs (Table 2).

### 2.5. Comparison of Total Phenolic (TP) and Total Flavonoid (TF) Contents in SWR and SBR

**Figure 5:**
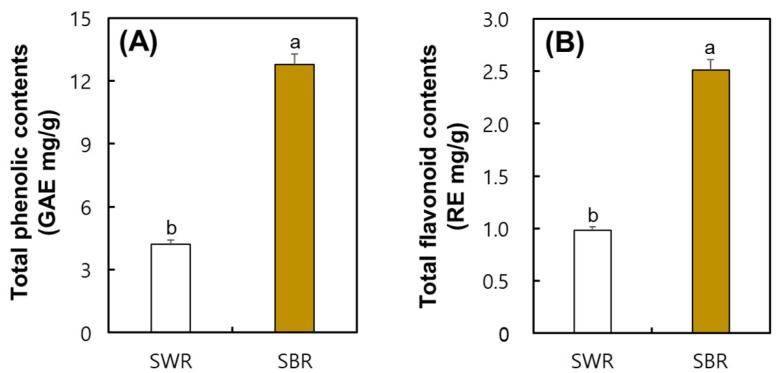
Comparison of total phenolic and flavonoid contents of the produced soybean-white roots and soybean-brown roots in a hydroponic cultivation system. (**A**) Total phenolic contents and (**B**) total flavonoid contents. All values are presented as the mean ± SD of pentaplicate determination. Different letters correspond to the significant differences relating to samples using Duncan’s multiple range tests (*p* < 0.05).

The comparison of TP and TF contents of soybean root extracts of white and brown color roots was depicted. As seen in Figure 5, the SWR and SBR groups revealed TP contents of 4.23 mg/g and 12.77 mg/g, respectively, indicating that the SBR sample exhibited approximately 3-fold greater TP contents. Furthermore, the TF contents in the SBR sample were estimated at 2.51 mg/g, indicating significantly higher TF contents than in the SWR group, which were 0.98 mg/g.

### 2.6. Comparison of Radical Scavenging Activities in SWR and SBR

**Figure 6:**
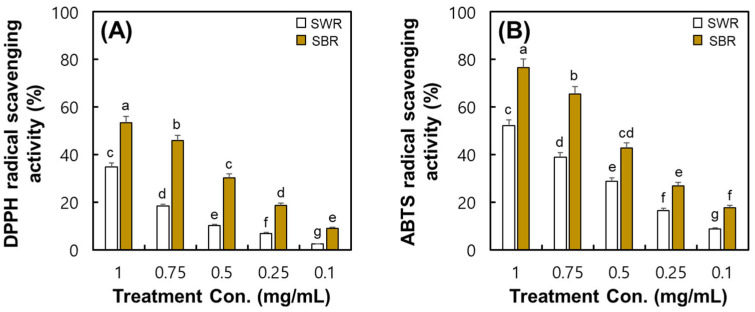
Comparison of radical scavenging activities on the produced soybean-white roots and soybean-brown roots in a hydroponic cultivation system. (**A**) DPPH radical scavenging activity and (**B**) ABTS radical scavenging activity. All values are presented as the mean ± SD of pentaplicate determination. Different letters correspond to the significant differences relating to samples using Duncan’s multiple range tests (*p* < 0.05).

The DPPH and ABTS radical scavenging activity of soybean root extracts is shown in Figure 6. At 1 mg/mL of root extract, the DPPH radical scavenging activity of SWR and SBR samples was estimated at 34.77% and 53.32%, respectively. At the same concentration of root extract, the ABTS radical scavenging activity of SWR and SBR samples was estimated at 52.04% and 76.44%, respectively. These results indicated the differences in the DPPH and ABTS radical scavenging activity of soybean roots according to color change, and the SBR samples exhibited greater radical scavenging activity in soybean roots. According to Table S1, the DPPH radical scavenging activity of SWR and SBR samples was equivalent to 12.46 mg/g and 18.94 mg/g ascorbic acid, respectively. Likewise, the ABTS radical scavenging activity of the SWR and SBR samples was equivalent to 19.06 mg/g and 31.96 mg/g Trolox, respectively.

## 3. Discussion

In legumes such as soybean, alfalfa, and mung beans, *Rhizobium* sp. establishes root nodules that provide the host plant with fixed nitrogen, hence enhancing growth and production [19]. In fact, plant growth-promoting rhizobacteria (PGPR) improve plant productivity by promoting root development, suppressing pathogen infestations, and boosting stress tolerance [11,20,21]. Accordingly, several studies indicate that bacterial interactions on plants may improve soybean nutritional and functional qualities, supporting sustainable agriculture and the production of biofunctional resources [22]. Such effects are likely mediated through microbial modulation of phytohormonal balance and metabolic fluxes, ultimately enhancing the biosynthesis of health-promoting compounds [23]. In this study, SWR outperformed SBR in terms of root length and shoot growth, suggesting that the presence of rhizospheric bacteria may have an impact on root development. Research has consistently reported that PGPR enhances root development in a variety of plant species [10,21]. In particular, *Bacillus subtilis* and *P. fluorescens* are well-reported inoculants that cause tomato plants to produce auxin and cytokinin, which causes the roots to elongate and branch laterally [24]. In contrast, under abiotic and biotic stress conditions, root development is impeded, since plants frequently increase the production of specific secondary metabolites as a survival strategy, rather than prioritizing growth, activating defense mechanisms [25]. For instance, environmental stress factors such as temperature, drought, and pathogen infection have been documented to reduce plant biomass while significantly promoting the synthesis of phenolic compounds [26]. This phenomenon is significant because it suggests that the potential for isoflavone accumulation may be influenced by environmental factors and the distribution of rhizospheric bacteria, and could be related to the inhibition of root development observed in SBR.

The SBR exhibited significantly higher concentrations of isoflavones, particularly daidzein derivatives, compared to SWR. This implies that interactions between bacteria in the rhizosphere may set off stress reactions that stimulate the biosynthesis of isoflavone [2,3,27]. For instance, *B. japonicum* promotes root nodule formation in legumes, which raises the expression of isoflavone synthase, a key enzyme in the isoflavone biosynthesis pathway, leading to the accumulation of isoflavones [28]. *Rhizobium* sp. and *Bacillus* sp. have been reported to promote secondary metabolite biosynthesis in legumes, with higher isoflavone accumulation upon bacterial inoculation [29]. Additionally, plant–microbe interactions have been demonstrated to induce the biosynthesis of flavonoid compounds, a process that reinforces plant defense mechanisms [27,30]. 16S rRNA gene sequencing revealed the higher abundances of genera such as *Brevundimonas*, *Paracoccus*, and *Delftia* in SBRs. These bacteria were reported to potentially induce the activation of a defense mechanism. For example, *D. acidovorans* strongly adheres to rapeseed roots and demonstrates the ability to establish root colonization during early plant development [31]. This process is largely regulated by plant–microbe signaling networks, where specific bacterial strains could activate stress response pathways to enhance the production of plant defense metabolites [25,32]. Therefore, these bacteria are believed to control root metabolic pathways, encouraging isoflavone production, which may account for the elevated isoflavone concentration observed in SBRs. The TP and TF contents were demonstrably higher in SBR, likely attributable to the bacterial stimulation of polyphenol biosynthetic pathways [33,34]. It is well-established that rhizosphere bacterial interactions regulate polyphenol metabolism, which in turn reinforces plant defense responses through the modulation of stress-related signaling pathways [25]. These findings emphasize the potential of plant–microbe symbioses in enhancing environmental adaptability and optimizing the production of functional metabolites, including antioxidants.

In accordance with these results, antioxidant activity assays utilizing DPPH and ABTS methods indicated that SBR displayed significantly higher radical scavenging activity than SWR. This activity is strongly correlated with the elevated levels of TP and TF in SBR, thereby supporting the hypothesis that these metabolites play a central role in the plant’s oxidative stress response system [35]. Prior studies have demonstrated that regulation of rhizospheric bacteria enhances antioxidant defense mechanisms by modulating the expression of antioxidant enzymes, such as superoxide dismutase, catalase, and peroxidase, thus improving the plant’s capacity for free radical scavenging [36]. Furthermore, specific bacterial inoculations have been documented to augment antioxidant enzyme activity under abiotic stress conditions, including drought and salinity, thereby contributing to plant resilience and growth in adverse environments [30]. These effects are typically mediated through rhizospheric bacterial signaling networks that activate stress-responsive gene expression and the biosynthesis of secondary metabolites [26]. Consequently, the enhanced antioxidant activity observed in SBR can likely be ascribed to both the increased accumulation of TP and TF compounds and the bacterial-mediated activation of antioxidant pathways. In addition to their well-documented role in direct radical scavenging, TP and TF compounds have been shown to modulate intracellular redox homeostasis and signaling pathways that mediate adaptive stress responses in plants [35,36]. In support of this assertion, our previous studies using soybeans have also demonstrated a significant correlation between increased isoflavone content and enhanced antioxidant activity [37,38]. Based on these findings, the current root-focused survey further corroborates that elevated TP, TF, and isoflavones in SBR samples may indicate both metabolic reprogramming and regulation by rhizospheric bacteria. Collectively, these findings underscore the critical role of cultivation environment in regulating plant metabolism and the distribution of rhizospheric bacterial communities.

**Figure 7:**
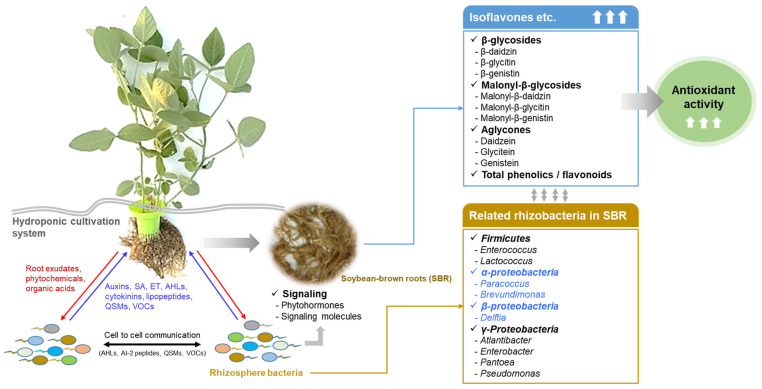
Mechanism of active metabolites (isoflavones) production by interaction between soybean roots and rhizobacteria. Soybean roots and rhizobacteria exhale compounds beneficial to each other to establish mutual relationships. Abbreviations: salicylic acid (SA), ethylene (ET), N-acyl homoserine lactones (AHL), quorum sensing molecules (QSM), and volatile organic compounds (VOCs).

Overall, this study demonstrates that SBRs exhibited significantly higher concentrations of isoflavones, TP, and TF than SWRs, alongside superior antioxidant activity. These results imply that intricate signaling and interactions between rhizosphere bacteria and plants may have activated secondary metabolic pathways and fortified defense mechanisms. Notably, the accumulation of functional substances, such as isoflavones, TP, and TF, may have influenced the increased relative abundance of *Paracoccus*, *Brevundimonas*, and *Delftia*, which could positively influence radical scavenging activity (Figure 7). A significantly different trend in microbial dominance was identified when compared with soil-based soybean cultivation systems reported in previous studies. In soil environments, the root-associated microbiota is generally dominated by genera such as *Burkholderia, Rhizobium*, and *Streptomyces*, which are recognized for their contributions to nitrogen fixation, pest control, and plant growth promotion [39]. However, these findings were neither prevalent nor detected in the current hydroponic NFT system, likely attributable to the absence of soil-derived microbiota and the selective pressures inherent in hydroponic cultivation [40]. This comparison underscores the considerable influence of the cultivation environment on the bacterial community composition and, consequently, root-associated metabolic processes. Notably, the increased production of isoflavones and antioxidant compounds may have contributed to the greater relative abundance of α- and β-proteobacteria observed in the SBR (Figure 7). However, long-term monitoring of bacterial dynamics and metabolite fluctuations is essential to comprehensively elucidate plant–microbe interactions [25,41]. Research tracking bacterial and metabolite dynamics will significantly enhance our understanding of these interactions [27].

## 4. Materials and Methods

### 4.1. Plant Materials, Reagents, and Equipment

The soybean seeds (Pungsan) were collected from the Korea Partnership for Innovation of Agriculture (KOPIA, Jeonju, Republic of Korea) in 2021 and subsequently stored at 4 °C for future use in experiments. The soybean plants were irrigated in a hydroponic system using Mulpure liquid (No.1 A, pH 6.0, EC 1.0 dS·m^–1^, Daeyu, Gyeongsan, Republic of Korea). Various reagents, including 5-sulfosalicylic acid, 2 N-Folin Ciocalteu phenol reagent, diethylene glycol, β-daidzine, β-glycitin, β-genistin, daidzein, glycitein, genistin, and 2,4,6-azino-bis (3-ethylbenzothiazoline-6-sulfonic acid) (ABTS), were purchased from Sigma-Aldrich (St. Louis, MO, USA). Additionally, standard compounds malonyl-β-daidzine, malonyl-β-glycitin, and malonyl-β-genistin were purchased from LC Laboratories (Woburn, MA, USA). Organic solvents, including water, acetonitrile (ACN), and methanol (MeOH), were purchased from J. T. Baker (Phillipsburg, NJ, USA). The DNA polymerase chain reaction (PCR) amplification was performed using an Eppendorf Vapo Protect Mastercycler Pro S 6325 system (Hamburg, Germany). Next, the 16S rRNA gene sequence was analyzed using an automated DNA sequencer (Applied Biosystems 3730 and 3730xl DNA Analyzers, Foster City, CA, USA). Isoflavone compounds were analyzed using high-performance liquid chromatography (HPLC, Agilent 1200 series, Agilent Co., Forest Hill, Vic, Australia), and TP, TF, and radical scavenging activity were measured using a spectrophotometer (UV-1800 240 V, Shimadzu Corp., Kyoto, Japan).

### 4.2. Plant Growth Conditions by Hydroponic Cultivation

Soybean plants were cultivated using the NFT technique, among other hydroponic methods. Initially, soybean seeds were subjected to a quenching process for 12 ± 1 h. Subsequently, one soybean seed was planted in each of the plastic cells. The plants were then transported to an NFT hydroponic machine (W × L × H, 10 × 95 × 10 cm; 6 holes per field) and placed inside the greenhouse. After that, 80 L of sterilized water was placed in the liquid reservoir for a week, supplied by a pump (Ultra power electric water pump UP200, Hyub Shin, Republic of Korea), replaced with 80 L of new sterilized water again, and 200 mL of liquid was diluted and circulated through the pump to the plant. The mean temperature of the greenhouse was 25 ± 3 °C, the mean humidity was 70 ± 5%, and harvests were performed at the R1 stage of soybean plants after 35 days of cultivation. The classification of soybean roots was tagged as SWRs and SBRs according to the color of the roots after harvest (Figure 1).

### 4.3. Isolation and Identification of Rhizospheric Bacteria

The bacterial isolation medium used in this experiment consisted of 30.0 g of tryptic soy broth (TSB, Difco, Becton Dickinson Co., Sparks, MD, USA) dissolved in 1 L of distilled water, along with 15 g of agar. The rhizospheric bacteria were isolated from the surface of hydroponically cultivated soybean roots. After adding 10-fold sterile distilled water to the samples of SWR and SBR, they were appropriately diluted. Then, spread onto TSB agar medium and incubated at 30 °C for 48 ± 2 h. Soybean root bacteria were then morphologically isolated, and inoculated into TSB medium containing 20% glycerol, and stored at –70 °C until further use. Identification of SWR and SBR was conducted according to the standard protocol of Lee et al. [42] with modifications. The TSB medium (5 mL) was inoculated with bacteria at 30 °C and 600 nm to achieve an OD > 0.5, while shaking the culture at 125 rpm. After transferring to a sterile microcentrifuge tube, the cultures were centrifuged for 5 min at 15,000× *g*. Following the manufacturer’s instructions, total DNA was isolated using the I-genomic BYF DNA Extraction Mini Kit (iNtRON, Seongnam, Republic of Korea). The isolated total DNA was amplified for PCR using bacteria-specific 16S rRNA oligonucleotides (877F, 5′-CGAGTTGATCCTG-3′; 878R, 5′-TACGCTTGTAC-3′) and Go Taq^®^ Green Master Mix (Promega Corp., Madison, WI, USA). PCR conditions included 40 cycles of 30 s at 94 °C for denaturation, 30 s at 49 °C for annealing, 90 s at 72 °C, with a 10 min extension at 72 °C. The 16S rRNA products were then detected using agarose gel electrophoresis, and the amplicons were purified with the MEGA quick-spin™ Total Fragment DNA Purification Kit (iNtRON). An automated DNA sequencer was employed to identify the 16S rRNA gene sequence. Bioinformatics analysis involved comparing the similarity between our obtained DNA sequences and other existing bacterial 16S rRNA sequences in the GenBank database (http://www.ncbi.nlm.nih.gov/Genbank; accessed on 8 October 2024).

### 4.4. Preparation of Sample Extracts

The soybean root was dried at 55 °C for 2 days using a dry oven (WOF-W155, DAIHAN Scientific Co., Ltd., Wonju, Republic of Korea) and stored in a deep freezer at –70 ± 1 °C. Dried samples (0.5 g) were placed in 50% MeOH (10 mL) and shaken for 24 ± 1 h at 25 °C ± 3. After centrifuging the extract for 3 min at 3000× *g*, the supernatant was filtered using a 0.45 μm membrane filter (Whatman plc., Little Chalfont, Buckinghamshire, UK) to assay the concentrations of isoflavones, TP, TF, and radical scavenging activities.

### 4.5. Determination of Isoflavone Contents in Soybean Root Extracts

The isoflavone analysis was performed according to the procedure of Lee et al. [42]. The HPLC instrument used in the study was equipped with an Agilent Co. (Santa Clara, CA, USA) Diode Array Detector from the Agilent 1260 series and LiChrospher 100 RP-18 columns (4.6 × 250 mm, 5 μm, Merck, Darmstadt, Germany). The parameters for the analysis were as follows: an injection volume of 20 μL, a column temperature of 30 °C, and an absorbance of 254 nm. The analytical solvents included water with 0.2% acetic acid (solvent A) and ACN containing 0.2% acetic acid (solvent B). For solvent B, the gradient conditions were as follows: at 15 min, 0–10%; at 25 min, 10–20%; at 35 min, 20–25%; at 45 min, 25–35%; at 50 min, 35%. The chemicals in the samples were identified by comparing the retention times to the relevant standards. With a coefficient of determination (R^2^) value greater than 0.998, the isoflavones were measured using linear calibration curves for each standard. Five unique standard points (6.25, 12.5, 25, 50, and 100 μg/mL) were used for the calibration curves.

### 4.6. Determination of TP Contents in Soybean Root Extracts

The TP contents of soybean roots were assayed according to the slight modifications to the method detailed by Lee et al. [42]. The extract (500 μL) was dispensed into test tubes, followed by adding 25% Na_2_CO_3_ (500 μL), and incubated for 3 min. Next, color development was performed at 37 °C, 1 h after the addition of 2 N Folin–Ciocalteu phenol reagent (250 μL). Afterward, the experimental sample was measured at an absorbance of 750 nm. The concentration of TP contents was expressed using the gallic acid (100, 50, 25, 12.5, and 6.25 μg/mL) standard curve.

### 4.7. Determination of TF Contents in Soybean Root Extracts

The TF contents of soybean root extracts were determined with a slight modification of the method of Lee et al. [42]. The 500 μL of diluted extract was distributed into a test tube, and 1000 μL of diethylene glycol and 10 μL of 1 N-NaOH were added thereto. After incubation in a 37 °C thermostat for 1 h, the experimental sample was measured at an absorbance of 420 nm. The TF contents were measured according to the standard curve made using a pure compound rutin (100, 50, 25, 12.5, and 6.25 μg/mL).

### 4.8. Determination of Radical Scavenging Activities of Soybean Root Extracts

#### 4.8.1. Analysis of DPPH Radical Scavenging Activity

The DPPH radical scavenging activity of the extracts was measured according to the protocol established by Jeong et al. [43]. This activity was determined by mixing 50% MeOH extracted samples (200 μL) with a 1.5 × 10^–4^ M DPPH solution (800 μL), inverting the mixture for 5 s, and allowing it to react for 30 min. The absorbance of the final reaction product was measured at 525 nm. In this experiment, a negative control test was conducted using 50% MeOH. The difference in absorbance values between the experimental and negative controls was calculated, and the DPPH radical scavenging activity was expressed as a percentage (%).

#### 4.8.2. Analysis of ABTS Radical Scavenging Activity

The ABTS radical scavenging activity of soybean root extract was measured with slight modifications to previously reported methods, following the protocol established by Jeong et al. [43]. This activity was measured by combining 7 mM ABTS (2.5 mL) and 2.45 mM K_2_S_2_O_8_ (7.5 mL) to form ABTS^+^ in the dark for 12 ± 2 h. Afterward, the ABTS^+^ solution was diluted with MeOH to achieve an absorbance value of 0.8 ± 0.02 at 732 nm. The sample extract (100 μL) was then mixed with the ABTS+ solution (900 μL) and allowed to react in the dark for 3 min. The absorbance of the final reaction product was measured at 732 nm. In this experiment, a negative control was conducted using 50% MeOH, and the difference in absorbance values between the experimental and negative controls was calculated. The ABTS radical scavenging activity was then expressed as a percentage (%):

Radical scavenging activity (%) = {1 − (A/B)} × 100,(1)

where “A” is experimental absorbance values and “B” is negative control absorbance values.

### 4.9. Statistical and Data Processing

Each study’s results were based on the mean ± standard deviation (SD) of pentaplicate measurements. ANOVA was performed using SAS software (version 9.4, SAS Institute, Cary, NC, USA), which showed statistically significant differences between the data, and Duncan’s multiple tests (*p* < 0.05) were used in order to confirm the findings. Plots were performed with the R studio server using the ‘corrplot’ package.

## 5. Conclusions

This study demonstrates that the growth environment significantly impacts the composition of functional metabolites in soybean plants and influences the structure of the rhizosphere bacterial community when using NFT, with the isoflavone compositions in the roots exhibits more considerable variation than in the upper tissues, with the total isoflavone contents accumulated by up to 3.9-fold (from 2095.16 to 8197.26 µg/g) in SBRs. In addition, the TP and TF contents were significantly higher in SBR. These changes were associated with enhanced radical scavenging activity, highlighting that the increased content of functional metabolites in soybean roots can influence the composition of the rhizosphere bacterial community. Therefore, these results demonstrate that the NFT system not only allows for the utilization of roots beyond the limits of soil cultivation but also shows potential as an enhanced source of bioactive compounds from rhizosphere bacteria. Future studies should validate these effects by co-inoculating isolated rhizospheric bacteria and utilize integrated multi-omics approaches, such as volatile organic compounds, metabolomics, microbiomics, and gene expression analysis, to uncover the mechanisms of microbe-induced metabolite regulation in soybean roots. In particular, a more comprehensive analysis of isoflavone subclasses and the gene expression related to flavonoid biosynthesis will aid in elucidating the direct role of specific microbial interactions in enhancing antioxidant functionality.

## Figures and Tables

**Table 1:** Comparison of isoflavone contents in organs of soybean plants, focusing on the produced soybean-white roots and soybean-brown roots in a hydroponic cultivation system.

Contents ^1^ (μg/g)	Hydroponic Cultivation System					
	Roots		Leaves		Stems	
	SWR	SBR	SWR	SBR	SWR	SBR
Glycosides						
Daidzin	93.39 ± 8.76 ^d^	1608.13 ± 14.01 ^a^	535.14 ± 66.88 ^b^	486.46 ± 58.98 ^b^	153.20 ± 6.82 ^c^	150.65 ± 9.00 ^c^
Glycitin	30.28 ± 1.01 ^b^	421.30 ± 5.02 ^a^	nd ^2^	nd	nd	nd
Genistin	18.08 ± 0.66 ^c^	92.17 ± 1.82 ^b^	320.96 ± 31.85 ^a^	338.95 ± 22.83 ^a^	10.05 ± 0.95 ^e^	13.83 ± 0.52 ^d^
Total	141.75	2121.59	856.10	825.41	163.25	164.49
Malonyl-β-glycosides						
Malonyl-β-daidzin	70.31 ± 1.42 ^b^	662.29 ± 8.60 ^a^	355.77 ± 41.69 ^c^	428.03 ± 68.12 ^b^	189.70 ± 2.80 ^e^	255.77 ± 3.84 ^d^
Malonyl-β-glycitin	21.85 ± 1.22 ^b^	146.76 ± 3.48 ^a^	nd	nd	nd	nd
Malonyl-β-genistin	35.36 ± 1.50 ^d^	121.40 ± 5.38 ^b^	601.11 ± 35.01 ^a^	607.32 ± 20.21 ^a^	96.14 ± 6.41 ^c^	129.80 ± 11.09 ^b^
Total	127.52	930.45	956.88	1035.35	285.84	385.56
Aglycones						
Daidzein	1636.0 ± 5.28 ^b^	4035.27 ± 9.33 ^a^	65.49 ± 2.99 ^d^	188.43 ± 0.81 ^c^	63.90 ± 1.89 ^d^	57.64 ± 2.08 ^d^
Glycitein	105.93 ± 4.75 ^b^	558.21 ± 13.05 ^a^	nd	nd	nd	nd
Genistein	83.96 ± 1.61 ^c^	551.73 ± 13.84 ^a^	34.13 ± 0.65 ^d^	92.80 ± 0.72 ^b^	16.55 ± 0.81 ^e^	13.97 ± 1.96 ^e^
Total	1825.90	5145.21	99.61	281.23	80.45	71.61
Sum of isoflavones	2095.16	8197.26	1912.59	2141.99	529.54	621.66

^1^ All values are presented as the mean ± SD of pentaplicate determination. Different letters correspond to the significant differences relating to the same row using Tukey’s multiple tests (*p* < 0.05). ^2^ nd, not determined.

**Table 2:** 16S rRNA gene sequence similarities of the isolated rhizosphere bacteria from produced soybean roots (white and brown roots) in a hydroponic cultivation system.

Isolates	Phylum	Result of NCBI Search (Accession No.) ^1^	Identity (%)	Cell Numbers (log cfu/g)
Soybean-white roots				
SWR01	*γ-Proteobacteria*	*Stenotrophomonas maltophilia* C2-6 (KY910070)	99	7.88
SWR02	*Firmicutes*	*Bacillus subtilis* ZIM3 (MT539995)	100	7.7
SWR03	*γ-Proteobacteria*	*Pseudomonas putida* HN2013 (MT515799)	99	9.16
SWR04	*γ-Proteobacteria*	*Pseudomonas putida* TS312 (AP022324)	99	9.67
SWR05	*Firmicutes*	*Enterococcus casseliflavus* AdM3 (MN213350)	97	9.22
SWR06	*γ-Proteobacteria*	*Stenotrophomonas maltophilia* OsEnbHZG5 (MN889390)	99	8.57
SWR07	*γ-Proteobacteria*	*Enterobacter asburiae* FDAARGOS1056 (CP066278)	99	8.97
SWR08	*γ-Proteobacteria*	*Pseudomonas mosselii* R10 (DQ073452)	100	7.88
SWR09	*γ-Proteobacteria*	*Pseudomonas fulva* Z67zhy (AM411071)	99	9.59
SWR10	*γ-Proteobacteria*	*Klebsiella michiganensis* CCMMB1233 (MW303474)	99	8.82
SWR11	*γ-Proteobacteria*	*Acinetobacter baumannii* 10B0D1-C1 (MN250320)	99	7.7
SWR12	*γ-Proteobacteria*	*Acinetobacter rhizosphaerae* Atecer9I (MT386200)	100	8.98
SWR13	*γ-Proteobacteria*	*Acinetobacter baumannii* APP173 (MT544603)	99	7.82
SWR14	*γ-Proteobacteria*	*Pseudomonas guariconensis* QH16-20 (MT078617)	99	7.4
SWR15	*Firmicutes*	*Lysinibacillus* sp. QT417 (MT033087)	100	7.4
SWR16	*γ-Proteobacteria*	*Stenotrophomonas maltophilia* AS1 (MT291866)	99	7.4
SWR17	*Firmicutes*	*Bacillus* sp. C7-7 (KY910114)	99	7.4
Soybean-brown roots				
SBR01	*γ-Proteobacteria*	*Klebsiella* sp. NTA-4 (MK886623)	98	9.86
SBR02	*γ-Proteobacteria*	*Pseudomonas* sp. JG 10 (EU937753)	99	11.5
SBR03	*γ-Proteobacteria*	*Pantoea* sp. NJ-32 (AM421978)	99	10.1
SBR04	*γ-Proteobacteria*	*Acinetobacter* sp. KL1 (GU566317)	99	9.97
SBR05	*Firmicutes*	*Enterococcus gallinarum* GI13 (MT158590)	100	10.26
SBR06	*γ-Proteobacteria*	*Atlantibacter hermannii* H2 (MK544072)	99	10.27
SBR07	*γ-Proteobacteria*	*Acinetobacter soli* IILSFSP371 (MN082084)	99	9.97
SBR08	*γ-Proteobacteria*	*Pseudomonas* sp. LYX (MN598629)	99	10.72
SBR09	*γ-Proteobacteria*	*Pseudomonas fulva* Z67zhy (AM411071)	99	10.41
SBR10	*Firmicutes*	*Enterococcus gallinarum* ZX1-1 (MG694659)	99	10.18
SBR11	*Firmicutes*	*Lactococcus lactis* 4355 (MT645510)	99	9.57
SBR12	*γ-Proteobacteria*	*Enterobacter* sp. AN3 (MT557019)	99	8.7
SBR13	*γ-Proteobacteria*	*Pseudomonas* sp. ANA71 (HQ219902)	99	10.71
SBR14	*γ-Proteobacteria*	*Pseudomonas* sp. PN-F1 (FJ463405)	99	9.57
SBR15	*γ-Proteobacteria*	*Pseudomonas oleovorans* PR51-16 (EU440977)	99	10.53
SBR16	*γ-Proteobacteria*	*Pseudomonas* sp. Z62zhy (AM410621)	99	10.78
SBR17	*γ-Proteobacteria*	*Acinetobacter* sp. MemClNew (KJ920201)	99	10.26
SBR18	*γ-Proteobacteria*	*Pseudomonas* sp. KFWK (MT397006)	99	9.21
SBR19	*α-Proteobacteria*	*Paracoccus yeei* MGA26-2 (HM218000)	99	8.4
SBR20	*γ-Proteobacteria*	*Acinetobacter calcoaceticus* EH52 (GU339280)	99	8.4
SBR21	*γ-Proteobacteria*	*Enterobacter asburiae* PSB6 (HQ242719)	99	9
SBR22	*γ-Proteobacteria*	*Stenotrophomonas maltophilia* C2-6 (KY910070)	99	9.1
SBR23	*Firmicutes*	*Bacillus* sp. Ce.BL.R.3 (MT126515)	98	9.18
SBR24	*β-Proteobacteria*	*Delftia tsuruhatensis* D9 (MT374262)	99	8.7
SBR25	*γ-Proteobacteria*	*Stenotrophomonas acidaminiphila* B4 (MN904905)	99	9.6
SBR26	*α-Proteobacteria*	*Brevundimonas* sp. 19D2A5 (MN620407)	99	8.4

^1^ Accession number of the nearest relative. When more than one sequence had the same similarity value, only the accession number of the first sequence is given.

## Data Availability

Data will be made available on request.

## Supplementary Materials

The following supporting information can be downloaded at: https://www.mdpi.com/article/10.3390/plants14142238/s1, Figure S1. Biomass amounts for different organs of the produced soybean-white roots and soybean-brown roots in the hydroponic cultivation system; Figure S2: Morphological characteristics (colony shape) on TSA media of 17 isolated rhizobacteria derived from soybean-white roots; Figure S3: Morphological characteristics (colony shape) on TSA media of 26 isolated rhizobacteria derived from soybean-brown roots; Table S1. Comparison of radical scavenging activity of the produced soybean-white roots and soy-bean-brown roots in a hydroponic cultivation system.

## Abbreviations

The following abbreviations are used in this manuscript:

ABTS	2,4,6-Azino-Bis (3-Ethylbenzothiazoline-6-Sulfonic acid)
ACN	Acetonitrile
DPPH	2,2-diphenyl-1-picrylhydrazyl
HPLC	High-Performance Liquid Chromatography
NFT	Nutrient Film Technique
PCR	Polymerase Chain Reaction
SWR	Soybean-White Root
SBR	Soybean-Brown Root
TF	Total Flavonoid
TP	Total Phenolic
TSB	Tryptic Soy Broth
