# Supplementary material for: Rhizospheric Bacterial Distribution Influencing the Accumulation of Isoflavones, Phenolics, Flavonoids, and Antioxidant Activity in Soybean Roots Within Hydroponic System
Source: Plants (Basel). 2025 Jul 19;14(14):2238. doi: 10.3390/plants14142238 (PMC12299990; doi:10.3390/plants14142238)
Supplement: Supplementary file 1 [file plants-14-02238-s001.zip › plants-3672923-supplementary.pdf]

Supplementary materials

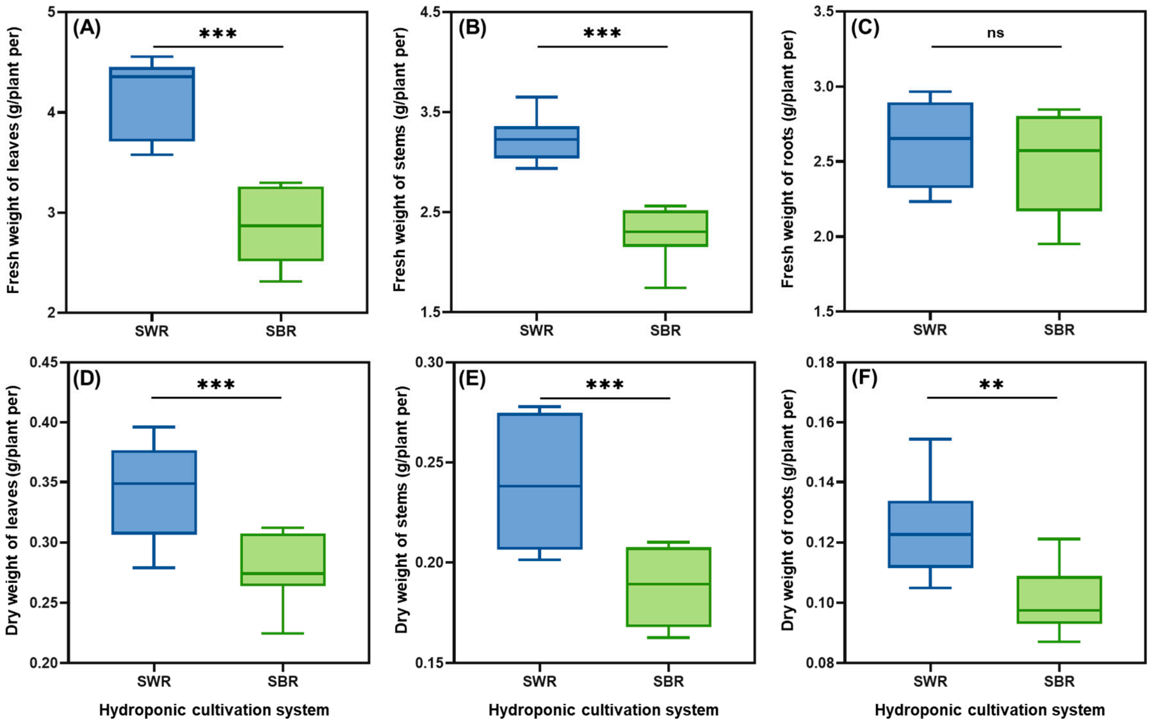

Figure S1. Biomass amounts for different organs of the produced soybean-white roots and soybean-brown roots in the hydroponic cultivation system. (A) Fresh weight of leaf; (B) fresh weight of stem; (C) fresh weight of root; (D) dry weight of leaf; (E) dry weight of stem; (F) dry weight of root. All values are expressed as the mean ± SD (n=10). Significant differences were determined between SWRs and SBRs using an unpaired t-test (ns, no significant; $*$, $p < 0.05$; $**$, $p < 0.01$; $***$, $p < 0.005$).

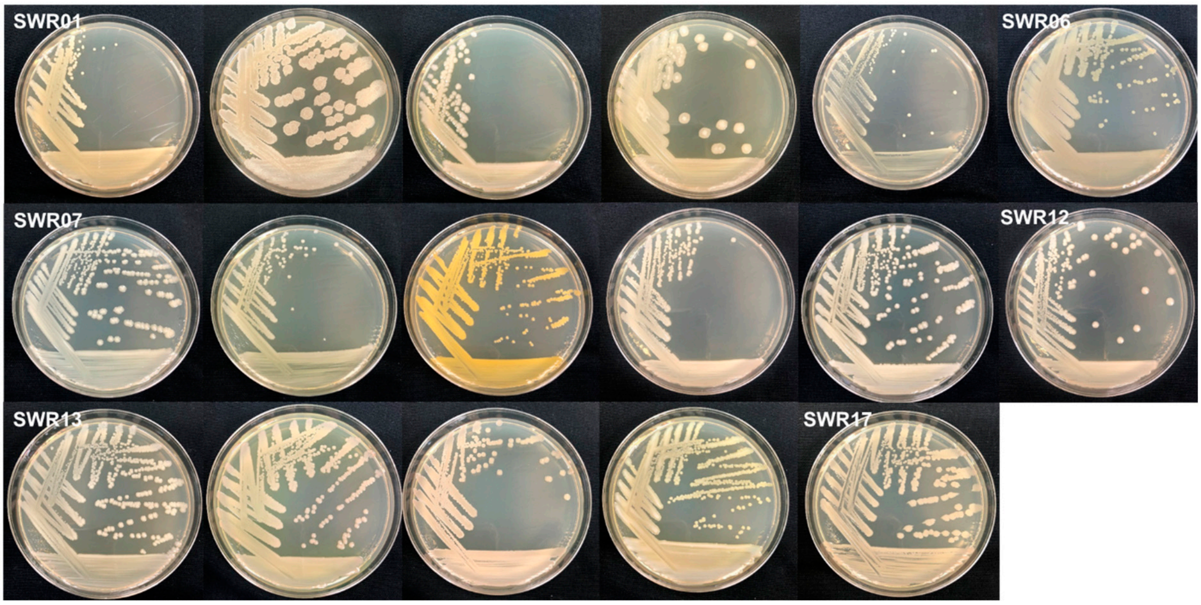

Figure S2. Morphological characteristics (colony shape) on TSA media of 17 isolated rhizobacteria derived from soybean-white roots.

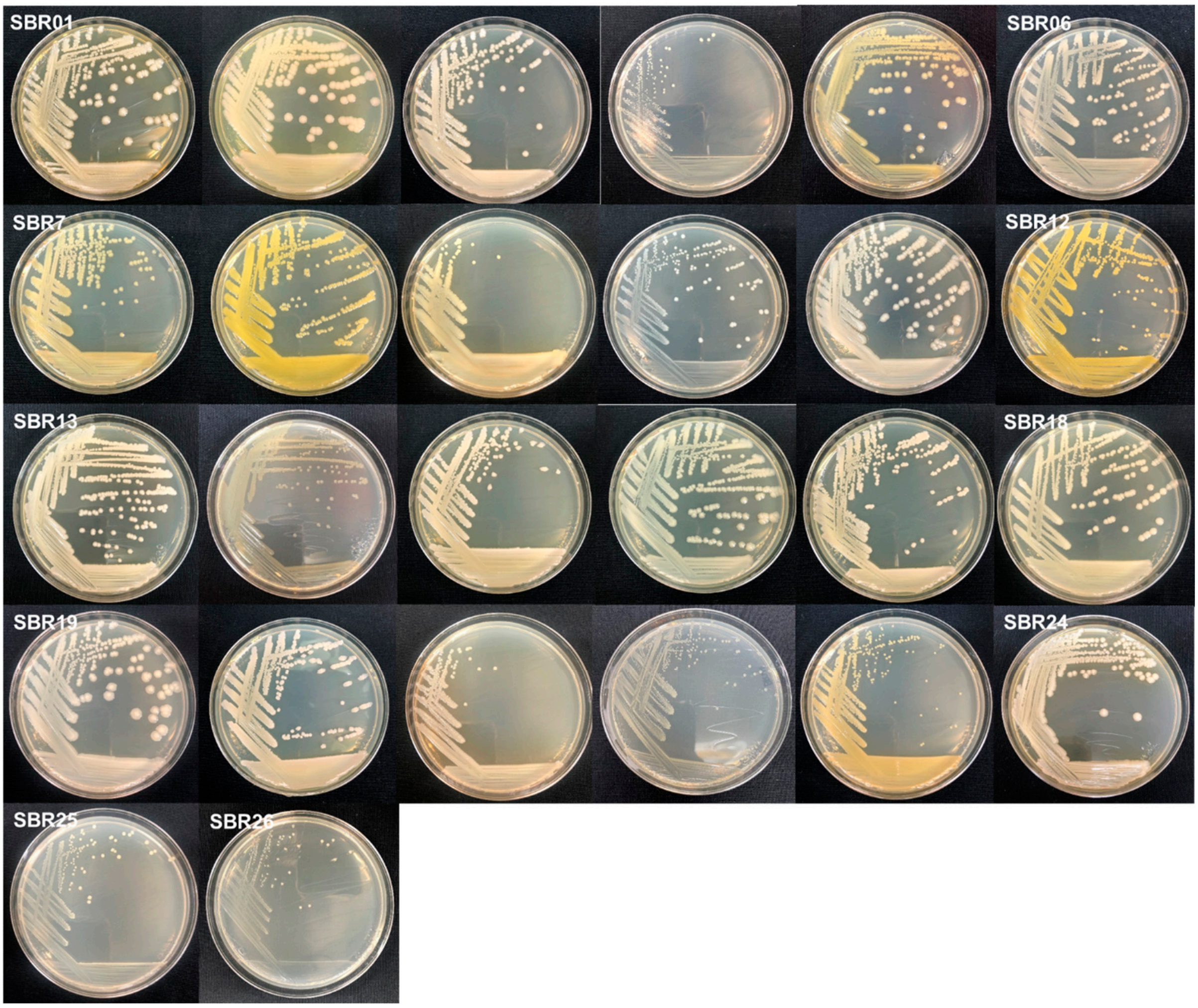

Figure S3. Morphological characteristics (colony shape) on TSA media of 26 isolated rhizobacteria derived from soybean-brown roots.

Table S1. Comparison of radical scavenging activity of the produced soybean-white roots and soybean-brown roots in a hydroponic cultivation system.

Index[1]	Hydroponic cultivation system	
	Soybean-white roots	Soybean-brown roots
DPPH radical scavenging activity (Ascorbic acid mg/g)	12.46 ± 0.66 [b]	18.94 ± 0.39 [a]
ABTS radical scavenging activity (Trolox mg/g)	19.06 ± 0.84 [b]	31.96 ± 0.40 [a]

[1] All values are presented as the mean ± SD of pentaplicate determination. Different letters correspond to the significant differences relating to samples using a t-test ($p < 0.05$).
